# NNLO QCD predictions for Z-boson production in association with a charm jet within the LHCb fiducial region

**DOI:** 10.1140/epjc/s10052-023-11530-x

**Published:** 2023-04-27

**Authors:** R. Gauld, A. Gehrmann–De Ridder, E. W. N. Glover, A. Huss, A. Rodriguez Garcia, G. Stagnitto

**Affiliations:** 1 Max Planck Institute for Physics, Föhringer Ring 6, 80805 München, Germany; 2 Institute for Theoretical Physics, ETH, 8093 Zürich, Switzerland; 3 Department of Physics, University of Zürich, 8057 Zürich, Switzerland; 4 Institute for Particle Physics Phenomenology, Durham University, Durham, DH1 3LE UK; 5 Department of Physics, Durham University, Durham, DH1 3LE UK; 6 Theoretical Physics Department, CERN, 1211 Geneva 23, Switzerland

## Abstract

We compute next-to-next-to-leading order (NNLO) QCD corrections to neutral vector boson production in association with a charm jet at the LHC. This process is studied in the forward kinematics at $\sqrt{s}=13$ TeV, which may provide valuable constraints on the intrinsic charm component of the proton. A comparison is performed between fixed order and NLO predictions matched to a parton shower showing mutual compatibility within the respective uncertainties. NNLO corrections typically lead to a reduction of theoretical uncertainties by a factor of two and the perturbative convergence is further improved through the introduction of a theory-inspired constraint on the transverse momentum of the vector boson plus jet system. A comparison between these predictions with data will require an alignment of a flavour-tagging procedure in theory and experiment that is infrared and collinear safe.

## Introduction

The study of scattering processes that involve the direct production of (heavy)-flavoured jets, i.e. those consistent with originating from charm (${\textrm{c}}$) or bottom (${\textrm{b}}$) quarks, in association with a leptonically decaying vector boson is essential for collider physics phenomenology. They form a major background for several Standard Model (SM) physics processes, including the production of a Higgs boson in association with a gauge boson where the Higgs boson decays into heavy-flavoured jets [1–5], as well as signals expected in models of physics beyond the SM (BSM) [6, 7]. Furthermore, they can provide unique information on the distribution of flavoured partons inside the proton [8–10]. Focussing on the process of ${\textrm{Z}}$ plus flavoured jet at the Large Hadron Collider (LHC), several measurements have been performed by the ATLAS, CMS and the LHCb collaborations at 7 and 8 TeV proton-proton collision energies [11–16]. Recent studies at 13 TeV by the CMS collaboration [17] presented measurements of observables related to the production of ${\textrm{c}}$ and/or ${\textrm{b}}$-quark jets in a sample containing a ${\textrm{Z}}$-boson produced in association with at least one jet.

The production of a leptonically decaying ${\textrm{Z}}$-boson in association with a charm jet, and particularly at forward kinematics [18] which is the focus of this work, could yield a unique probe of the charm content of the proton [18–20], provided that precise predictions and measurements of flavour-sensitive ${\textrm{Z}} +{c}\text {-jet}$ observables are available and can be compared at a similar level of accuracy. The LHCb collaboration has recently analysed events containing a ${\textrm{Z}}$-boson and a charm jet in the forward region of phase space in proton-proton collisions [9]. These measurements simultaneously provide direct access to the small- and large-*x* regions of the ${\textrm{c}}$-quark parton distribution function (PDF) that is not well explored by other experiments. Specifically, LHCb has presented results [9] for the ratio of production cross sections $R^{c}_{j} = \sigma ({\textrm{Z}} +{c}\text {-jet})/\sigma ({\textrm{Z}} +\text {jet})$. This ratio is measured differentially as a function of the rapidity of the ${\textrm{Z}}$-boson $y^{{\textrm{Z}}}$ in the range $2.0< y^{{\textrm{Z}}}<4.5$. The experimental result for the ratio $R^{c}_{j}$ has been compared with several SM predictions obtained at NLO QCD accuracy interfaced with a parton shower (NLO+PS), each using different input PDF sets. It is demonstrated that the most forward $y^{{\textrm{Z}}}$ region is particularly sensitive to the theoretical modelling of the charm quark PDF in these sets, with the best agreement between theory and data obtained by choosing a PDF set with a valence-like intrinsic (non-perturbative) charm quark component. The presence or absence of this component is a long standing theoretical issue [21, 22]. Recently, the NNPDF collaboration has claimed evidence for an intrinsic charm quark component in the proton [23], with a local significance at the 2.5$\sigma$ level for momentum fractions in the region $0.3 \lesssim x \lesssim 0.6$. By including the LHCb data for $R^{c}_{j}$ in a reweighting of their fit, and adopting a theory prediction based on NLO+PS, the local significance further increases to about 3.0$\sigma$. Other PDF fitting collaborations have independently investigated the possible presence of intrinsic charm in the proton [24]: for instance, a recent analysis by the CTEQ-TEA collaboration [25] concludes that finding evidence for nonperturbative charm continues to be elusive, by highlighting challenging aspects that must be confronted in extracting nonperturbative charm in PDF fits.

State-of-the-art predictions for this kind of processes featuring the associated production of a vector boson with one or more flavoured jets has reached next-to-next-to-leading order (NNLO) accuracy in QCD calculations with massless quarks [26–28]. Given the importance of the LHCb data for PDF extractions, it is highly desirable to have fixed-order predictions for the ${\textrm{Z}} +{c}\text {-jet}$ process in the forward region, in order to incorporate data for $R^{c}_{j}$ or other flavour-sensitive observables in global PDF analyses based on collinear factorisation. In this paper we focus on a description of ${\textrm{Z}} +{c}\text {-jet}$ production within the LHCb fiducial region following the approach of [29] to define flavoured jets, and provide a detailed comparison of (new) fixed-order predictions up to NNLO QCD accuracy as well as those based on NLO+PS for a variety of differential distributions.

Despite our focus on the forward kinematics relevant to the LHCb detector, in this work we refrain from performing a comparison to the available LHCb data [9]. This is due to a significant contamination of the observable $R^{c}_{j}$ measured by LHCb from Multiple Particle Interactions (MPI), which should be removed/subtracted before considering this data in a (single parton scattering) collinear PDF fit. Moreover, the experimental definition of jet flavour in [9] is not infrared and collinear (IRC) safe, rendering a massless fixed-order calculation ill defined for the experimental set-up. With the goal in mind to provide constraining information on the potential presence of an intrinsic charm quark component within the proton, it is critical that the definition of the presented data is IRC safe such that a massless calculation (where collinear factorisation for the charm-quark PDF has been performed) can be applied. We elaborate more on these issues in Appendices A and B.

The structure of the paper is as follows. In Sect. 2, we present the main ingredients entering the computation of observables associated with ${\textrm{Z}} +{c}\text {-jet}$ production both at pure fixed-order perturbative QCD as well as using the NLO+PS framework. We further comment on the proposal of [29], the *flavour dressing* algorithm, which allows flavour to be assigned to anti-$k_{\textrm{T}}$ jets in an IRC safe way. In Sect. 3, after having described the numerical set-up and defined the scale variation prescriptions, we present for the first time fixed-order predictions up to next-to-next-to-leading order (NNLO) in QCD for several observables related to the ${\textrm{Z}} +{c}\text {-jet}$ process computed for the LHCb experimental fiducial region at 13 TeV. We compare these fixed-order predictions with NLO predictions matched to a parton shower at the parton level using the flavour dressing procedure to define flavoured jets. We further investigate the impact of an additional constraint on the transverse momentum of the vector boson plus jet system in the computations. We shall find that the inclusion of this theoretically motivated cut, brings NNLO and NLO+PS predictions closer and improves the perturbative convergence of the fixed-order results for a large fraction of the flavour-sensitive observables and for most of the kinematical range studied. We also present predictions for the ratio $R^{c}_{j}$. In Sect. 4, we summarise our findings and discuss the prospects of a direct comparison between theory and data in the future. The Appendices contain a discussion of the IRC safety of the current experimental definition (Appendix A) and the role of MPI in the current experimental set-up (Appendix B).

## Details of the calculation

In this paper, one of the main goals is to present fixed-order predictions including QCD corrections up to $\mathcal{O}(\alpha _{s}^{3})$ for observables related to the process ${\textrm{p}} {\bar{\textrm{p}}} \rightarrow {{\ell }} {{\bar{\ell }}} +{c}\text {-jet} + X$, yielding a final state that contains a pair of charged leptons and at least one identified charm jet.

The computation of higher order corrections to observables with identified flavour poses several challenges as compared to flavour-blind cross sections: it requires a complete flavour tracking of the particles in all subprocesses (which are inputs to the flavour-dependent jet reconstruction/tagging), and additionally to those appearing in all subtraction terms (for a calculation based on subtraction). A flavour tracking procedure at parton level has been pioneered for the computation of b-jet flavoured observables in [30] and [26] and implemented within the NNLOjet parton-level generator that is used here. Within this framework, which employs the antenna subtraction method to capture the infrared behaviour of matrix-elements yielding fully differential cross section predictions at NNLO level, the flavour tracking procedure has the crucial property that it can be applied to any flavour-blind computation already present in the NNLOjet code. For example, the computation of ${\textrm{Z}} +{b}\text {-jet}$ observables in [26] relied on the use of the existing flavour blind ${\textrm{Z}} +\text {jet}$ computation presented in [31] and used the flavour-$k_{\textrm{T}}$ algorithm to select b-jets. To compute observables related to the ${\textrm{Z}} +{c}\text {-jet}$ production process in this work, we adopt a similar strategy by using the ${\textrm{Z}} +\text {jet}$ computation including up to $\mathcal{O}(\alpha _\textrm{s} ^3)$ corrections, and then apply the flavour tracking procedure as was done for the ${\textrm{Z}} +{b}\text {-jet}$ process.

As was the case in [26], the prediction of scattering processes with heavy-flavour jets can be further improved by exactly including the contribution from a massive heavy-flavour quark at fixed order—i.e. the resultant prediction is made in a general mass variable flavour number scheme. We follow a similar procedure here and include mass corrections up to $\mathcal{O}(\alpha _{s}^{2} m_{{\textrm{c}}})$ in the fixed-order distributions which are labelled as NLO and NNLO.

A further complication which is encountered in a calculation of the type presented here—the calculation of QCD corrections to a scattering process with flavoured jets based on massless quarks—is that an IRC safe definition of jet flavour must be used. A first solution to this problem was introduced in [32], with the formulation of the IRC safe flavour-$k_{\textrm{T}}$ algorithm. This algorithm features a $k_{\textrm{T}}$-like clustering sequence, and introduces a specific flavour-dependent clustering sequence to achieve IRC flavour safety. However, since the algorithm requires the knowledge of the flavour of all the particles in the event at each step of the clustering, it is challenging to realise experimentally, and so far has not been implemented in experimental analyses. Therefore, the kinematics and flavour of the jets obtained with the flavour-$k_{\textrm{T}}$ algorithm are not compatible with those obtained in experiment. Various alternatives to the use of flavour $k_T$ in theoretical predictions have recently been proposed [33–35].

In the present analysis, we will adopt the flavour dressing algorithm of [29]. This approach is particularly suitable as it enables to assign flavour quantum numbers to a set of flavour blind jets, obtained with any jet clustering algorithm. This allows us to apply it to jets reconstructed with the anti-$k_{T}$ algorithm [36], i.e. the same algorithm which is used in experiment to define the kinematics of the jets (although the flavour assignment procedure is different, as detailed in Appendix A). Here, we stress the fact that in the flavour dressing algorithm the flavour assignment is entirely factorised from the initial jet reconstruction, hence the kinematics of the jets is not affected. Such a key property is relevant in the present context, since it ensures that for a ratio observable such as $\sigma ({\textrm{Z}} +{c}\text {-jet})/\sigma ({\textrm{Z}} +\text {jet})$ both the numerator and the denominator feature the same sample of anti-$k_{\textrm{T}}$ jets.

A comparison of the fixed-order predictions as described above will also be made to several NLO predictions matched with a parton shower. Those NLO+PS predictions are obtained either with the MadGraph5_aMC@NLO (v. 2.7.3) [37] framework interfaced to Pythia8 (v. 8.243) [38] (default $p_{\textrm{T}}$-ordered parton shower) or Herwig7 (v. 7.2.2) [39–41] (default angular-ordered parton shower), and using the same flavour dressing procedure to define flavoured jets as for the fixed-order predictions. To allow for a more direct comparison, those predictions are obtained at the parton level where neither the impact of MPI or hadronisation effects are included. A discussion on the important role of MPI for the ${\textrm{Z}} +{c}\text {-jet}$ process in the forward region is provided in Appendix B. Hadronisation effects, on the other hand, were found not to impact the considered observables in any significant manner.

## Numerical results

### Numerical set-up and scale variation prescription

In this section, we review the calculational set-up as well as the kinematical constraints imposed to obtain the fiducial cross sections for Z+${c}\text {-jet}$ production. To select our final-state events, we focus on the forward region with fiducial cuts mirroring those of the LHCb measurement [9] at $\sqrt{s}=13$ TeV.

In particular, the following fiducial cuts for jets and charged leptons are applied: $20~\textrm{GeV}< p_{\textrm{T},j} < 100~\textrm{GeV}$, $2.2< \eta _{j} < 4.2$, $p_{\textrm{T},\ell } > 20~\textrm{GeV}$, $2.0< y_{\ell } < 4.5$, $M_{\ell \bar{\ell }} \in [60,120]~\textrm{GeV}$ and $\varDelta R(j,\ell ) > 0.5$. The jets are reconstructed with the anti-$k_T$ algorithm [36] with $R=0.5$. As discussed in Sect. 2, the selection of ${c}\text {-jets}$ is performed using the flavour dressing procedure described in [29]. The algorithm proceeds in two stages with internal parameters that control the overall flavour-tagging procedure: a flavour clustering stage that employs a Soft-Drop-inspired criterion [42] with parameters $z_\textrm{cut}$, $R_\textrm{cut}$, and $\beta$, followed by a flavour dressing stage based on the flavour-$k_T$ distance measure [32] with a parameter $\alpha$. In the present calculation, we set the parameters to their default values [29]: $z_\textrm{cut} = 0.1$, $R_\textrm{cut} = 0.1$, $\beta = 2$, $\alpha = 2$. In addition, events are only retained if the flavour-tagged ${c}\text {-jet}$ is the jet carrying the largest transverse momentum of reconstructed jets passing the selection cuts.

**Fig. 1:**
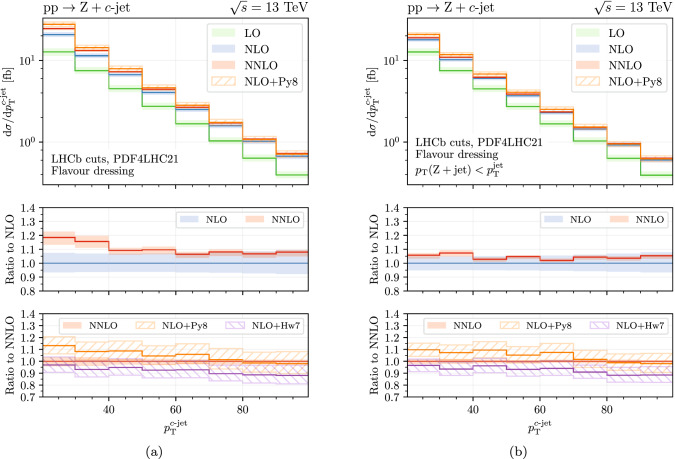
Comparison of parton-level predictions for the leading flavoured jet transverse momentum $p_{\textrm{T}}^{{c}\text {-jet}}$ in the ${\textrm{Z}} +{c}\text {-jet}$ process: fixed-order predictions at LO (green), NLO (blue) and NNLO (red); NLO+PS predictions with Pythia8 (orange) or Herwig7 (purple) as parton showers. A dynamical cut on the transverse momentum of the ${\textrm{Z}} +\text {jet}$ system is further applied in **b**

We provide predictions for proton-proton collisions at $\sqrt{s}=13$ TeV and use the PDF4LHC21 Monte Carlo PDF set [43], with $\alpha _s(M_{\textrm{Z}}) = 0.118$ and $n_f^\textrm{max} = 5$, where both the PDF and $\alpha _s$ values are accessed via LHAPDF [44]. For the electroweak input parameters, the results are obtained in the $G_{\mu }$-scheme, using a complex mass scheme for the unstable internal particles, and we adopt the following values for the input parameters: $M_{{\textrm{Z}}}^\textrm{os} = 91.1876~\textrm{GeV}$, $\varGamma _{{\textrm{Z}}}^\textrm{os} = 2.4952~\textrm{GeV}$, $M_{{\textrm{W}}}^\textrm{os} = 80.379~\textrm{GeV}$, $\varGamma _{{\textrm{W}}}^\textrm{os} = 2.085~\textrm{GeV},$ and $G_\mu = 1.1663787 \times 10^{-5}~\textrm{GeV} ^{-2}$.

**Fig. 2:**
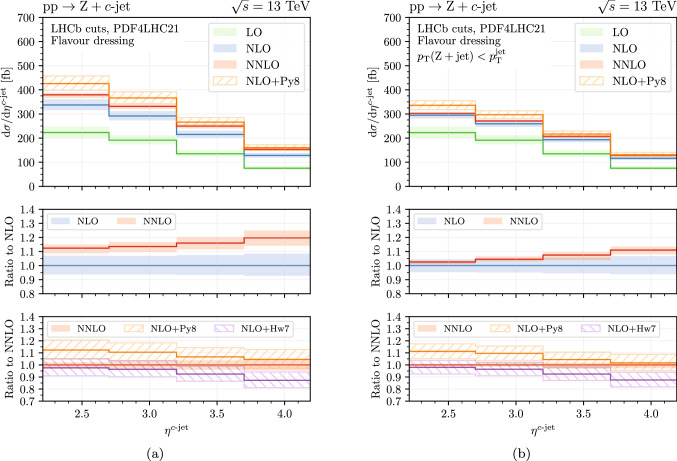
Comparison of parton-level predictions for the leading flavoured jet pseudo-rapidity $\eta ^{{c}\text {-jet}}$ in the ${\textrm{Z}} +{c}\text {-jet}$ process: fixed-order predictions at LO (green), NLO (blue) and NNLO (red); NLO+PS predictions with Pythia8 (orange) or Herwig7 (purple) as parton showers. A dynamical cut on the transverse momentum of the ${\textrm{Z}} +\text {jet}$ system is further applied in **b**

**Fig. 3:**
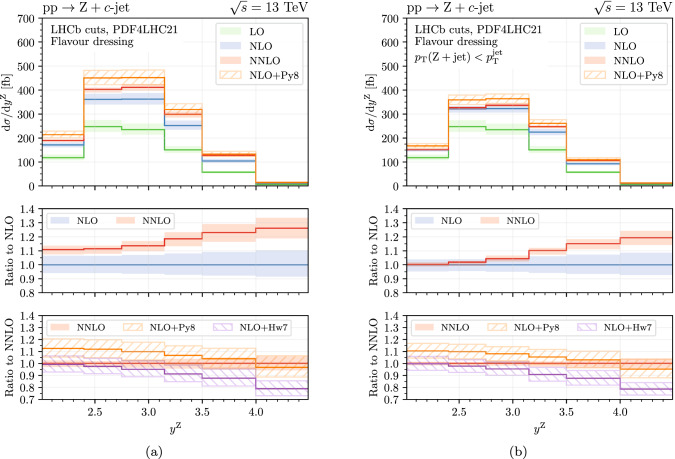
Comparison of parton-level predictions for the rapidity distribution of the lepton pair $y^Z$ in the ${\textrm{Z}} +{c}\text {-jet}$ process: fixed-order predictions at LO (green), NLO (blue) and NNLO (red); NLO+PS predictions with Pythia8 (orange) or Herwig7 (purple) as parton showers. A dynamical cut on the transverse momentum of the ${\textrm{Z}} +\text {jet}$ system is further applied in **b**

For differential distributions, the impact of missing higher-order corrections is assessed using the conventional 7-point scale variation prescription: the values of factorisation ($\mu _F$) and renormalisation ($\mu _R$) scales are varied independently by a factor of two around the central scale $\mu _0 \equiv E_{\textrm{T},{\textrm{Z}}}$, with the additional constraint that $\frac{1}{2} \le \mu _F/\mu _R \le 2$.

When considering theoretical predictions for the ratio of distributions, we estimate the uncertainties in an uncorrelated way between the numerator and denominator i.e. by considering

1 $$\begin{aligned} R^{c}_{j} (\mu _R,\mu _F;\mu '_R,\mu '_F) = \frac{\sigma ^{{\textrm{Z}} +{c}\text {-jet}}(\mu _R,\mu _F)}{\sigma ^{{\textrm{Z}} +\text {jet}}(\mu '_R,\mu '_F)}, \end{aligned}$$

providing a total of 31-points when dropping the extreme variations in any pair of scales.

### $Z+c$-jet distributions

We here present results for the ${\textrm{Z}} +{c}\text {-jet}$ process at $\sqrt{s}=13$ TeV and choose to focus on the following observables: the leading flavoured jet transverse momentum $p_{\textrm{T}}^{{c}\text {-jet}}$ (Fig. 1), the leading flavoured jet pseudorapidity $\eta ^{{c}\text {-jet}}$ (Fig. 2), and the rapidity of the ${\textrm{Z}}$-boson $y^{{\textrm{Z}}}$, reconstructed from the two final-state opposite-charge leptons (Fig. 3). Besides presenting results for the LHCb kinematical set-up as indicated in Sect. 3.1, we also explore the impact of the introduction of a cut on the transverse momentum of the ${\textrm{Z}} +\text {jet}$ system:

2 $$\begin{aligned} p_{\textrm{T}}({\textrm{Z}} +\text {jet}) < p_{\textrm{T}}^{\text {jet}}, \end{aligned}$$

with the leading jet in the acceptance region. The theoretical motivation behind this cut is to discard those contributions where the flavoured jet is not the jet with the largest transverse momentum in the event, i.e. cases where the hardest jet was disregarded because it fell outside of the LHCb acceptance. At Born level, the $p_{\textrm{T}}$ of the ${\textrm{Z}} +\text {jet}$ system vanishes, hence the cut in (2) limits the hard QCD radiation outside the LHCb acceptance in a dynamical way.

For each of the figures presented in the remainder of this section, the left sides present results without the additional cut of (2), whereas the right sides present results where the additional cut has been imposed, such that the impact of the cut can be seen comparing the left and right hand sides of the figures. To highlight the size and shape of the fixed-order results at each perturbative order, and to best compare fixed-order with and without matching to PS results, all figures illustrating the results in this section are composed of three panels: the top-panel shows the absolute predictions at fixed-order (LO, NLO, NNLO) and for NLO+PS where PS is modelled by Pythia8; the middle panel shows the ratio of NNLO to the fixed-order NLO result while the lower panel shows the ratio to NNLO of the NLO+PS results where PS is modeled by either Pythia8 or Herwig7. As noted in Sect. 2, fixed-order predictions labelled as NLO and NNLO in all the figures include QCD corrections up to ${{\mathcal {O}}}(\alpha _{s}^{2})$ (NLO) and ${{\mathcal {O}}}(\alpha _{s}^{3})$ (NNLO) obtained with massless c-quarks in the computations, and both additionally include the exact charm-quark mass corrections up to ${{\mathcal {O}}}(\alpha _{s}^{2})$.

**Fig. 4:**
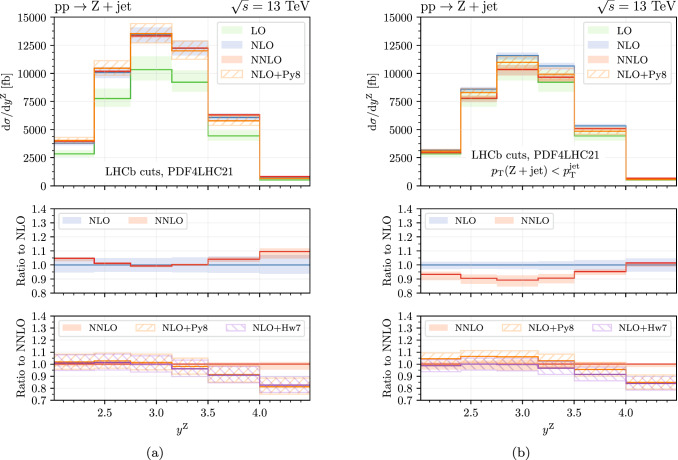
Comparison of parton-level predictions for the rapidity distribution of the lepton pair $y^Z$ for the unflavoured process ${\textrm{Z}} +\text {jet}$: fixed-order predictions at LO (green), NLO (blue) and NNLO (red); NLO+PS predictions with Pythia8 (orange) or Herwig7 (purple) as parton showers. A dynamical cut on the transverse momentum of the ${\textrm{Z}} +\text {jet}$ system is further applied in **b**

**Fig. 5:**
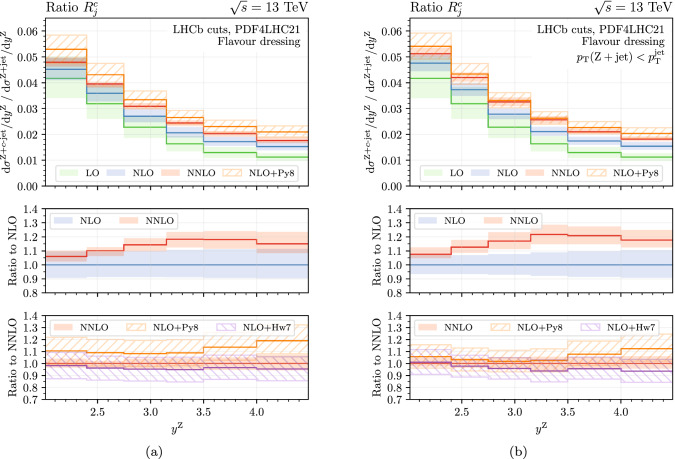
Comparison of parton-level predictions for the ratio $R^{c}_{j} = \sigma ^{{\textrm{Z}} +{c}\text {-jet}}/\sigma ^{{\textrm{Z}} +\text {jet}}$ differential in the rapidity $y^{{\textrm{Z}}}$ of the system of the final-state leptons: fixed-order predictions at LO (green), NLO (blue) and NNLO (red); NLO+PS predictions with Pythia8 (orange) or Herwig7 (purple) as parton showers. A dynamical cut on the transverse momentum of the ${\textrm{Z}} +\text {jet}$ system is further applied in **b**

The theory predictions which *do not* include the additional kinematic constraint of Eq. (2) are shown in Figs. 1a, 2a, and 3a. The NNLO corrections are observed to be of the order (10–20)% as compared to the NLO prediction, and typically outside of the scale variation band of the NLO result. In particular, the shape of the distribution is modified in the large-$\eta ^{{c}\text {-jet}}$ and large-$y^{{\textrm{Z}}}$ bins in Fig. 2a in Fig. 3a respectively, and in the small-$p_{\textrm{T}}^{{c}\text {-jet}}$ region in Fig. 1a, featuring a larger cross section at NNLO. Those features are made apparent in the second panel of those figures. In the third panel, the NNLO and both NLO+PS predictions are shown normalised to the central NNLO result. It is found that the NNLO result always lies between the two different NLO+PS results. We observe that the NNLO result is more consistent with the NLO+Herwig7 prediction for lower values of $p_{\textrm{T}}^{{c}\text {-jet}}$, $\eta ^{{c}\text {-jet}}$, and $y^{{\textrm{Z}}}$, and instead agrees better with NLO+Pythia8 predictions at larger values. Overall, the NLO+Herwig7 prediction seems to be rather similar to the NLO prediction, and the angular-ordered parton shower does not appear to impact flavour-sensitive observables in a significant way. Instead, the NLO+Pythia8 prediction does seem to capture some of the NNLO higher order corrections: at large-$p_{\textrm{T}}^{{c}\text {-jet}}$,$\eta ^{{c}\text {-jet}}$ and $y^{{\textrm{Z}}}$ values it tends to reproduce the shape of the fixed-order NNLO corrections.

The theory predictions that *do* include the additional kinematic constraint of Eq. (2) are shown in Figs. 1b, 2b, and 3b. We observe that the constraint leads to a slight reduction of the fiducial cross section and produces no significant change in the shape of the distributions with the exception of the low-$p_{\textrm{T}}^{{c}\text {-jet}}$ region. The LO, NLO, and NNLO results display an improved mutual compatibility across all considered observables, indicating a better perturbative convergence in the presence of this kinematic constraint. Qualitatively, the comparison between the NNLO and NLO+PS results in the third panel of these figures is similar to that of the case without the kinematic constraint which was already discussed.

Overall, for all considered set-ups, we find that the NNLO corrections bring a new level of precision for the considered ${\textrm{Z}} +{c}\text {-jet}$ observables. As compared to the corresponding NLO(+PS) results, the uncertainties of the NNLO predictions are reduced by a factor of two or more. It is also reassuring that the NNLO corrections lead to predictions that tend to lie between the two different NLO+PS predictions—the latter differ in the treatment of ${{\mathcal {O}}}(\alpha _{s}^{3})$ terms, which begin at the NNLO level. As the perturbative convergence of the predictions appears to be improved by applying the kinematic constraint of Eq. (2), with only a small reduction in the cross section, it seems to be well motivated when directly considering ${\textrm{Z}} +{c}\text {-jet}$ observables.

### The $\sigma ({\textrm{Z}} +\text {jet})$ cross-section and the ratio $R^{c}_{j}$

In Sect. 3.2 we have presented IRC safe predictions for the rates of ${\textrm{Z}} +{c}\text {-jet}$ production within the LHCb fiducial region. Instead, in this subsection we will consider the ${\textrm{Z}} +\text {jet}$ process (i.e. the flavour inclusive one) and then subsequently the ratio observable $R^{c}_{j} = \sigma ({\textrm{Z}} +{c}\text {-jet})/\sigma ({\textrm{Z}} +\text {jet})$. As the experimental measurement of $R^{c}_{j}$ is performed differentially in the rapidity of the ${\textrm{Z}}$-boson $y^{{\textrm{Z}}}$ [9], our theory predictions will also focus on this same quantity. We again note that the we do not perform any comparison to the available data for reasons of consistency, as detailed in Appendix A and B.

The theory predictions for ${\textrm{Z}} +\text {jet}$ production are presented in Fig. 4, with the same structure for the plots as shown in Sect. 3.2, i.e. with the “no-cut” and “with-cut” cases shown on the left and right parts of the figure respectively, and with three panels for each sub-figure. As compared to the ${\textrm{Z}} +{c}\text {-jet}$ predictions, heavy-flavour mass corrections have not been included for these predictions. While this could be achieved following the procedure outlined in [45], the numerical impact of such corrections for the flavour inclusive process is negligible (sub-percent).

The theory predictions without the extra kinematic cut of Eq. (2) are shown in Fig. 4a). The NNLO result is observed to be contained within the scale variation band of the NLO one, except in the large-$y^{{\textrm{Z}}}$ region, where it also features a different behaviour compared to the NLO+PS results. We also find very good agreement between the two NLO+PS results with Pythia8 and Herwig7.

The impact of applying the additional kinematic cut is shown in Fig. 4b. The cut leads to negative NNLO corrections in the (relatively) low $y^{{\textrm{Z}}}$ region, resulting in a NNLO prediction lying outside the scale variation band of the NLO result, except in the large-$y^{{\textrm{Z}}}$ region. Examination of the upper panel of Fig 4b shows that the cut has the effect of moving all the curves closer to the LO result, with positive NLO corrections and negative NNLO corrections. The inclusion of this cut thus appears to degrade the perturbative stability for the flavour-inclusive set-up.

We now consider the ratio observable $R^{c}_{j}$, differential in $y^{{\textrm{Z}}}$. The result is constructed using the same inputs which lead to the distributions for ${\textrm{Z}} +{c}\text {-jet}$ results in Fig. 3 and ${\textrm{Z}} +\text {jet}$ in Fig. 4, but including the uncorrelated uncertainty prescription defined in Eq. (1). The predictions for $R^{c}_{j}$ are displayed in Fig. 5, again the “no-cut” and “with-cut” cases are shown on the left and right sides of the figure respectively. Focussing first on the fixed-order results, the NNLO corrections are observed to be positive and of the order (10–20)% with the largest value observed at large-$y^{{\textrm{Z}}}$ values. That behaviour is observed for both the “no-cut” (left) and “with-cut” (right) cases. Overall, the inclusion of the cut on the ${\textrm{Z}} +\text {jet}$ system does not significantly impact the perturbative behaviour of the fixed-order prediction for the ratio. This is a consequence of the fact that the reduction of uncertainties in the numerator for ${\textrm{Z}} +{c}\text {-jet}$ is then compensated by increased uncertainties in the denominator, when the cut is applied. However, by inspecting the lowest panels of Fig. 5, we observe slightly better agreement between NNLO and the two NLO+PS predictions when the cut has been applied.

Finally, as a result of the uncorrelated prescription for uncertainties, we note that the relative theory uncertainties for the NNLO predictions of the ratio are increased as compared to the individual predictions for ${\textrm{Z}} +{c}\text {-jet}$ and ${\textrm{Z}} +\text {jet}$. The sensitivity to the input PDFs is also typically reduced for such an observable due to correlations between PDF-dependence of the numerator and denominator. With the aim of reducing the theory uncertainties due to missing higher corrections, one could therefore consider to include absolute ${\textrm{Z}} + {c}\text {-jet}$ cross-section data rather than that for the ratio $R^c_j$ in a collinear PDF fit. Given however that several experimental uncertainties are correlated between numerator and denominator (and therefore cancel in the ratio), and that a treatment of the MPI contribution to the observable should also be considered, overall it is not clear which observable is the most sensitive in constraining PDFs.

## Conclusions

In this paper, we have studied the associated production of a ${\textrm{Z}}$-boson with a charm-jet at the LHC at 13 TeV in the forward region. We computed NNLO predictions at $\mathcal {O}(\alpha _s^3)$ for a set of differential observables related to the ${\textrm{Z}} +{c}\text {-jet}$ process using the flavour dressing procedure to define charm-tagged jets. NNLO corrections are found to be at the level of (10–20)%; they can impact the shapes of distributions with the high-$y^{{\textrm{Z}}}$ and low-$p_{\textrm{T}}^{{c}\text {-jet}}$ regions receiving enhanced corrections. The residual uncertainties as estimated through scale variations are typically $\pm 5$% or smaller, a factor two reduction compared to the respective NLO uncertainty estimate. Additionally, comparisons to two different NLO+PS predictions based on the Herwig7 (angular ordering) and Pythia8 ($p_\textrm{T}$ ordering) showers have been performed. The two predictions can differ by up to 10% but remain mutually compatible within their respective uncertainties that are NLO-like and thus at the level of $\pm 10$%. The NNLO distributions are found to lie in between the two NLO+PS predictions, hinting to an insensitivity to missing higher-order effects as modelled by the showers. Moreover, we have found that a theory-inspired constraint on the transverse momentum of the ${\textrm{Z}} +\text {jet}$ system improves the perturbative convergence of all considered distributions.

We further considered the ratio $R^c_j = \sigma ^{{\textrm{Z}} +{c}\text {-jet}}/\sigma ^{{\textrm{Z}} +\text {jet}}$, which has been measured by the LHCb experiment. In this context, the usage of the flavour dressing procedure ensures the same kinematic reconstruction of jets entering the numerator and denominator, thus allowing for a faithful theoretical definition. The pattern of higher-order corrections mimics those of the ${\textrm{Z}} +{c}\text {-jet}$ process with enhanced NNLO corrections of up to 20% in the high-$y^{{\textrm{Z}}}$ region, albeit with larger uncertainties due to the de-correlated scale prescription for the ratio.

A direct comparison to the available LHCb data was not performed due to IRC unsafety issues and an unexpectedly large contamination from MPI; both of which are discussed in the Appendices. In the future, a fair comparison between experimental measurements and theory predictions will require a detailed study of the experimental feasibility of the flavour dressing algorithm (or other IRC safe variants). In the case a direct application of an IRC-safe flavour definition is prohibitively challenging, it would be highly desirable for experimental measurements to carry out an unfolding to a IRC-safe definition of jet flavour. Only a joint effort of both communities, theory and experimental, will enable to exploit in the best way the huge amount of data that LHC will provide us in the next decades, better enabling the use flavour signatures as a powerful window into short-distance interactions from $\textrm{GeV}$ to $\textrm{TeV}$ energy scales.

## Figures and Tables

**Fig. 6:**
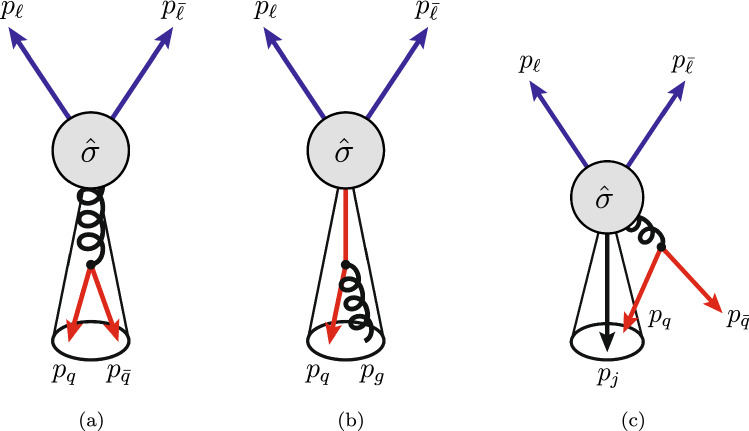
Examples of configurations which lead to an IRC sensitivity: **a** a double (even) tagged jet; **b** a tag with a $p_{\textrm{T},q}^\textrm{min}$ requirement; **c** a jet tag with a soft sensitivity (in the absence of a $p_{\textrm{T},q}^\textrm{min}$ requirement)

**Fig. 7:**
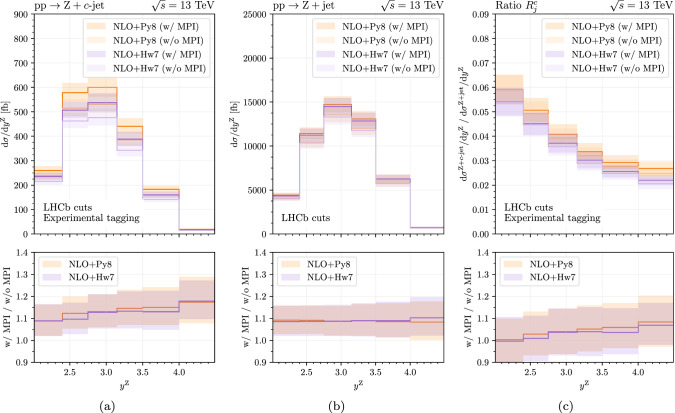
Effect of MPI contributions on the ${\textrm{Z}}$ rapidity distribution $y^{\textrm{Z}}$ in the ${\textrm{Z}} +{c}\text {-jet}$ process (**a**), in the ${\textrm{Z}} +\text {jet}$ process (**b**) and in the ratio of the two (**c**). NLO+PS predictions are obtained with Pythia8 (orange) or Herwig7 (purple) as parton showers. In the upper panels predictions including (excluding) MPI contributions are depicted in darker (lighter) colours. The lower panels show the ratios of curves with and without MPI effects

## Data Availability

This manuscript has no associated data or the data will not be deposited. [Authors’ comment: For this publication only computer-generated pseudo data were used. These have been obtained with either publicly available Monte Carlo event generators or with a private code.]

## Appendinx A: IRC safety

According to Ref. [9], as part of the LHCb measurement, charm jets are defined in the following way. First, jets are reconstructed using the anti-$k_T$ algorithm [36] with $R=0.5$, and the leading jet passing the fiducial selection is considered (see also Sect. 3.1 for the definition of the fiducial selection). From this, the leading jet is considered to be a charm jet (at truth/unfolded level) if it additionally satisfies the criterion: $p_{\textrm{T},c{~{\textrm{hadron}}}} > 5~\textrm{GeV}$, and $\varDelta R(j,c{~{\textrm{hadron}}}) < 0.5$. That is to say, the jet is considered to be charm tagged if there is **at least** one $c{~\mathrm hadron}$ satisfying these selections.

Such an experimental definition of jet flavour is collinear unsafe. This is not an LHCb specific issue (or even particular to this measurement), but is also relevant in one way or another for the definitions of jet flavour commonly adopted by LHC collaborations [46–49]. We briefly explain why the observable definition taken in Ref. [9] is IRC unsafe, then discuss the implications of this for data interpretation.

In Fig. 6 we depict three specific kinematic configurations which lead to different sources of IRC sensitivity (i.e. those which are IRC unsafe). In each case, the configurations correspond to those encountered in a parton-level fixed-order prediction for the ${\textrm{Z}} +{c}\text {-jet}$ process. The flavoured quarks are depicted as red lines, i.e. representing charm quarks for the process under consideration here.

- The first configuration depicts the production of the lepton pair, recoiling against a hard $q{\bar{q}}$ pair produced in a collinear configuration (i.e. at least one, or both, of the quarks are hard but $p_q \cdot p_{\bar{q}} \rightarrow 0$). When the anti-$k_{\textrm{T}}$ algorithm is applied, the *q* and ${\bar{q}}$ are reconstructed inside the same jet. According to the prescription to assign jet flavour, as this jet contains **at least** one quark it will be assigned a quark flavour tag (e.g. charm for $q = c$). This introduces a collinear sensitivity as it is distinguished from the case where the hard gluon does not split into a collinear $q\bar{q}$ pair. In that case, the jet (composed of a single hard gluon) clearly carries zero quark flavour. This could be overcome by accounting for the total quark flavour in the jet, such as assigning quantum numbers $q({\bar{q}})= +(-)1$ and summing them to obtain the net flavour (alternatively one can consider flavoured jets as those with an overall odd number of *q* and ${\bar{q}}$).
- The second configuration depicts the production of the lepton pair, recoiling against a hard *qg* pair in a collinear configuration (i.e. at least one, or both, of the quark and gluon is hard but $p_q \cdot p_{g} \rightarrow 0$). Again, when the anti-$k_{\textrm{T}}$ algorithm is applied, both *q* and *g* are reconstructed inside the same jet. As the tagging prescription requires the presence of a *c*-hadron with $p_{\textrm{T},c} > 5~\textrm{GeV}$, it is possible that the outgoing quark does not satisfy this criterion (depending on the momentum sharing with the gluon). This introduces a collinear sensitivity as the $p_{\textrm{T},c}$ requirement may distinguish from the case where the hard initial quark does not split to the collinear *qg* pair.
- The collinear sensitivity discussed above can be overcome by removing the $p_{\textrm{T},c}^\textrm{min}$ requirement. However, this would introduce a new problem which is depicted in the third configuration. In this case, the culprit is a soft gluon which subsequently splits to a $q{\bar{q}}$ pair at wide angles. It is possible that one of the quarks ($p_q$ in the figure) is produced close in $\varDelta R$ to a hard parton ($p_j$), i.e. $\varDelta R(j,q) < 0.5$. This would introduces a soft sensitivity as the flavour of the jet would be altered by the presence of the soft quark.

From a purely experimental point of view, it is clear that the current definition of tagging heavy-flavour jets is a sensible one. Identifying those jets with multiple tags (as oppose to at least one) requires to more carefully account for the experimental (in)efficiency and mistag rates. It is also extremely to difficult distinguish between the signature of one or two collinear heavy-flavour objects (e.g. a bunch of displaced tracks appearing to originate from a single displaced vertex). Furthermore, removing the $p_{\textrm{T},c}^\textrm{min}$ would mean accounting for a region where it is experimentally challenging to identify displaced vertices. However, this choice has serious ramifications for the theory predictions, and importantly for the interpretation of the data.

Theoretical predictions of charm jet observables which are not IRC safe are logarithmically sensitive to the mass of the charm quark $m_c$. The corresponding fixed-order predictions for such observables therefore include corrections which depend logarithmically on the charm quark mass. If the observable/process under consideration involves energy scales which are large compared to the quark mass (e.g. the transverse energy/momentum of a jet or a boson), the logarithmic corrections become large (due to the separation of scales) and thus limit the theory precision/perturbative stability. The $m_c\rightarrow 0$ limit of such predictions is not well defined (it is divergent), meaning that a calculation based on massless quarks of such observables does not exist. The implications of this are that fixed-order predictions must be performed in a scheme where the charm quark is massive, i.e. in a fixed-flavour-number scheme with $n_f^\textrm{max} = 3$, where mass factorisation is not performed for the charm quark, and it is decoupled from the running of $\alpha _\textrm{s}$. Note that the perturbative charm-quark PDF does still exist in the massive scheme (where a logarithmic sensitivity to the charm-quark mass exists, see for example [50–55]). Practically, it is generated numerically after integration over the phase-space of the massive quark during the calculation.

The requirement that a fixed-order prediction must be carried out with a massive calculation is problematic for observables which are designed to be sensitive to the nature of the charm quark PDF. Such observables contain a logarithmic sensitivity on the charm-quark mass as a result of the IRC unsafe configurations which were highlighted above (which limit the theory precision/predictability), at the same time the observables are (by design) directly sensitive to the large logarithmic corrections associated to the perturbative charm quark PDF which have not been resummed, and the non-perturbative component of the charm quark PDF (which is aiming to be probed) does not exist.

Instead, the definition of jet flavour used in this work, given its IRC safety, removes all logarithmic sensitivity on the charm quark mass which results from the jet flavour assignment procedure. This also allows for the application of a massless calculation, where mass factorisation for the charm quark is performed and where remaining logarithmic sensitivity to the quark mass is resummed.

## Appendix B: Multiple particle interactions

During the high-energy scattering of two protons, there is a probability for multiple hard-interactions to occur (i.e. more than one).

For the LHCb kinematics defined at the beginning of Sect. 3.1, and also applying the (IRC unsafe) definition of jet flavour as in [9], we observe a large contribution to the production of a ${\textrm{Z}}$ boson and a ${c}\text {-jet}$ from MPI. In Fig. 7a we show the cross-section for ${\textrm{Z}} +{c}\text {-jet}$ production after fiducial cuts, which is plotted differentially with respect to the ${\textrm{Z}}$-rapidity $y^{{\textrm{Z}}}$. The predictions are provided at NLO+PS accuracy for ${\textrm{Z}} +1j$ events generated with MadGraph5_aMC@NLO interfaced with Pythia8 and Herwig7, where the role of MPI is subsequently modelled by the two different Monte Carlo generators. We show the predictions obtained when including/excluding the MPI contributions, which lead to a large (and rapidity dependent) effect on the resultant distribution. In the central rapidity region, the effect is of order 10%, increasing up to 20% at larger rapidities, and the effect is very similar within Pythia8 and Herwig7. In Fig. 7b we further show the same plot for the unflavoured process ${\textrm{Z}} +\text {jet}$. We note a constant shift of the $y^{{\textrm{Z}}}$ distribution of order 10% due to MPI effects. The 10–20% effects observed in Fig. 7a for ${\textrm{Z}} +{c}\text {-jet}$ could be explained with an interplay of a constant 10% effect (impacting both ${\textrm{Z}} +{c}\text {-jet}$ and ${\textrm{Z}} +\text {jet}$ processes) and an additional 10% flavour-dependent effect. Finally, we consider the ratio $R^{c}_{j}$, even if its behaviour w.r.t. MPI effects can be straightforwardly inferred from Fig. 7a, b. In particular, we observe a partial cancellation of MPI effects, leading to a difference between predictions which is negligible at $y^{\textrm{Z}} \sim 2.0$ and that increases monotonically up to 10% at larger rapidities. Hence, given such a partial cancellation in the ratio, the inclusion of MPI effects seems to be particularly relevant for $3.5 \lesssim y^{\textrm{Z}} \lesssim 4.5$. Such a region is hugely important for the determination of the intrinsic charm in the proton, given that the LHCb measurement in the bin $y^{\textrm{Z}} \in [3.5, 4.5]$ is highly correlated to the charm PDF in the region of the valence peak $x \simeq 0.45$ [23].

As these MPI contributions arise from the overlap of multiple hard interactions, it is not taken into account by a theoretical description based solely on single parton scattering contributions (i.e. those which form the basis of a collinear PDF fit). If the data is to be considered for such a fit, it would be necessary to first remove/subtract the MPI component. Due to the complicated (overlapping) nature of multiple hard interactions, the removal of this component may rely on theoretical modelling. As the theoretical description of inclusive charm-quark production with the LHCb acceptance suffers from uncertainties far in excess of $50\%$ (see for example, Fig. 6 of Ref. [56] for $p_{\textrm{T}}^D \sim 5~\textrm{GeV}$), the modelling of this contribution (and the associated uncertainty) will require quite some care. We note that such an uncertainty (optimistically, 50% of the MPI contribution as shown in Fig. 7) would already be in excess of the systematic uncertainty quoted for the ratio observable in [9]. It may be possible to overcome this issue if a data-driven based approach can be achieved.
